# Neighbourhood walkability, road density and socio-economic status in Sydney, Australia

**DOI:** 10.1186/s12940-016-0135-y

**Published:** 2016-04-27

**Authors:** Christine T. Cowie, Ding Ding, Margaret I. Rolfe, Darren J. Mayne, Bin Jalaludin, Adrian Bauman, Geoffrey G. Morgan

**Affiliations:** South West Sydney Clinical School, UNSW Australia, Sydney, NSW Australia; Ingham Institute of Applied Medical Research, Sydney, NSW Australia; Woolcock Institute of Medical Research, University of Sydney, Sydney, NSW Australia; Prevention Research Collaboration, Sydney School of Public Health, University of Sydney, Sydney, NSW Australia; University Centre for Rural Health, University of Sydney, Lismore, NSW Australia; Public Health Unit, Illawarra Shoalhaven Local Health District, Wollongong, NSW Australia; Sydney School of Public Health, University of Sydney, Sydney, NSW Australia; Illawarra Health and Medical Research Institute, Wollongong, NSW Australia; Graduate School of Medicine, University of Wollongong, Wollongong, NSW Australia; South West Sydney Local Health District, Sydney, NSW Australia; School of Public Health and Community Medicine, UNSW Australia, Sydney, NSW Australia

**Keywords:** Walkability, Air pollution, Traffic, Neighbourhood, Transport, Health, Nitrogen dioxide

## Abstract

**Background:**

Planning and transport agencies play a vital role in influencing the design of townscapes, travel modes and travel behaviors, which in turn impact on the walkability of neighbourhoods and residents’ physical activity opportunities. Optimising neighbourhood walkability is desirable in built environments, however, the population health benefits of walkability may be offset by increased exposure to traffic related air pollution. This paper describes the spatial distribution of neighbourhood walkability and weighted road density, a marker for traffic related air pollution, in Sydney, Australia. As exposure to air pollution is related to socio-economic status in some cities, this paper also examines the spatial distribution of weighted road density and walkability by socio-economic status (SES).

**Methods:**

We calculated walkability, weighted road density (as a measure of traffic related air pollution) and SES, using predefined and validated measures, for 5858 Sydney neighbourhoods, representing 3.6 million population. We overlaid tertiles of walkability and weighted road density to define *“sweet-spots”* (high walkability-low weighted road density), and *“sour- spots*” (low walkability-high weighted road density) neighbourhoods. We also examined the distribution of walkability and weighted road density by SES quintiles.

**Results:**

Walkability and weighted road density showed a clear east-west gradient across the region. Our study found that only 4 % of Sydney’s population lived in *sweet-spot*” neighbourhoods with high walkability and low weighted road density (desirable), and these tended to be located closer to the city centre. A greater proportion of neighbourhoods had health limiting attributes of high weighted road density or low walkability (about 20 % each), and over 5 % of the population lived in *“sour-spot”* neighbourhoods with low walkability and high weighted road density (least desirable). These neighbourhoods were more distant from the city centre and scattered more widely. There were no linear trends between walkability/weighted road density and neighbourhood SES.

**Conclusions:**

Our walkability and weighted road density maps and associated analyses by SES can help identify neighbourhoods with inequalities in health-promoting or health-limiting environments. Planning agencies should seek out opportunities for increased neighbourhood walkability through improved urban development and transport planning, which simultaneously minimizes exposure to traffic related air pollution.

**Electronic supplementary material:**

The online version of this article (doi:10.1186/s12940-016-0135-y) contains supplementary material, which is available to authorized users.

## Background

The built environment, which refers to the totality of places designed and built by humans, plays an important role in population health [1, 2]. In the past few decades, increasing evidence suggests that aspects of the built environment are associated with health related behaviours such as physical activity [3], and health related outcomes such as obesity [4], cardiovascular health [2], and mental well-being [1]. A key concept is “walkability”, which encompasses built environment characteristics that are conducive to utilitarian walking (i.e. walking to destinations, including work commutes, errands, shopping), such as high residential density, good street connectivity, and land use mix [5]. Although walkability has been well studied in the context of physical activity, there is limited literature on its interaction with other key environmental attributes, such as outdoor air pollution [6].

Traffic related air pollution (TRAP) is a major contributor to ambient air pollution in most large cities, and comprises a complex mixture of primary and secondary particulate matter and gases such as oxides of nitrogen (NOx) [7–10]. For example, in Sydney, Australia, on-road vehicles contribute around 71 % of total NOx emissions [11]. Peak concentrations of NOx and nitrogen dioxide (NO_2_) occur near roads and there is evidence of pollutant decay within a few hundred metres distance from busy roads [12, 13]. Hence both NOx and NO_2_ are often used as markers of TRAP in epidemiological studies. Recent reviews have reported on associations between TRAP exposure and a range of adverse health outcomes including decreased lung function, increased airway inflammation, asthma symptoms, cardiovascular disease, hospitalisations, premature mortality and adverse birth outcomes [7, 8, 10, 14].

Methods for estimating exposure to TRAP at locations and spatial scales where air quality data or modelled estimates are not available include using measures of road proximity, traffic counts, traffic density (eg. traffic counts within certain radii around an address) or road density [10]. Advantages of these measures are that they are easily calculated if counts or a road classification system exists and they are inexpensive to implement. The added advantage of using traffic density measures is that they include the impact of a network of roads around a point of interest, and may better reflect exposure than a proximity measure [15]. A previous Sydney study found that a simple weighted road density (WRD) measure explained 59 % of the variance in NO_2_ measured by passive samplers, which was equivalent to the variability explained by using traffic density estimates using traffic count data, suggesting that WRD could be used as a proxy for exposure to TRAP [15].

The first and only study to date, as far as we are aware, to quantitatively estimate the spatial interaction between walkability and air pollution was conducted in Vancouver, Canada [6]. The authors calculated a four-factor walkability index [5] for each postal code and used a land use regression model to assign each postcode an annual average estimated nitrogen oxide (NO) concentration, as a marker of TRAP. This study found “trade-offs” between neighbourhood walkability and air pollution and identified a relatively small proportion of neighbourhoods that do especially well (high walkability and low TRAP - defined as *“sweet spots”*) and especially poorly (low walkability and high TRAP - defined as *“sour spots”*). Given the health consequences of both physical inactivity and exposure to TRAP, the Vancouver study highlighted the importance of characterising the complex spatial patterns of these two urban environmental health exposures, to guide transport policy and land use planning initiatives to maximize health gains.

A number of studies have characterised the relationship between socioeconomic characteristics of neighbourhoods and air pollution, with more deprived or disadvantaged areas often, but not always, exposed to higher air pollution concentrations [16–18]. This scenario is often termed a “double-burden of geography”. Not surprisingly, some studies have also found central or inner city advantaged areas to be subject to high air pollution concentrations, so the relationship is not always linear or in the expected direction [16, 19]. Aside from Marshall et al. [6] Vancouver study, none of these studies have investigated the relationship between SES and air pollution with respect to neighbourhood walkability.

The objectives of our study were to: 1) analyse the association between neighbourhood walkability and weighted road density (as a measure of traffic related air pollution) in the Sydney metropolitan area; and 2) describe the spatial distribution of these two characteristics, and 3) examine the relationship between neighbourhood socio-economic disadvantage and walkability/weighted road density.

## Methods

### Study area

We assessed the spatial distribution of WRD and walkability for 2007 in the Sydney metropolitan area. Sydney is Australia’s largest city, located on the eastern seaboard with an area of over 3700 km^2^ and had an estimated resident population of 3.6 million in 2006 (average density 990 persons/km^2^). Both walkability and WRD were calculated at the 2006 Australian Census Collection District (CCD) level, the smallest geographical area used for reporting Australian census aggregated household data by the Australian Bureau of Statistics (ABS) [20]. We obtained digital boundaries for CCDs from the ABS [20]. The average CCD in the study region included 200 dwellings and 550 residents and covered an area ranging from 0.002 km^2^ to 125.40 km^2^ (median 0.20 km^2^, 10^th^ percentile 0.02 km^2^, 90^th^ percentile 2.40 km^2^) prior to CCD exclusions due to missing data [21]. Usual resident population, percentage of total population, and land area (km^2^) were calculated for each CCD using data from the ABS 2006 Census Basic Community Profile DataPack (ABS cat no 2069.0.30.001). Percentage of people (employed adults aged at least 16 years) within each CCD walking entirely to work on the 2006 Census day was also calculated using data from the 2006 ABS Census.

### Measures

#### Walkability

We used an abridged walkability index previously developed for Sydney [21] that was modelled on the South Australian PLACE study index [22] and the walkability index developed by Frank et al in North America [5]. The Sydney walkability index includes the following three environmental attributes.

- residential density: the number of dwellings per square kilometre of residential land use;
- intersection density: the number of intersections with three or more road junctions per square kilometre of total land area. Intersection density is a measure of connectivity and is highest for streets with grid like patterns and lower for curvilinear street networks with long block lengths, with cul-de-sacs or with other boundaries such as motorways or railway lines.
- land use mix (the combination of five land classes adjusted for spatial area). Land use mix was calculated as the proportion of areas (km^2^) corresponding to specific land uses (residential, commercial, industrial, recreational, other) multiplied by the natural logarithm of the proportions. Scores ranged from 0–1 where 0 indicated a single land use and 1 indicated a mix of all 5 land use categories. The scores were then divided by the ratio of each CCD area compared to the smallest CCD area in the study region to adjust for differences in spatial scale [21].

Each of the three environmental attributes was then divided into deciles from 1 (lowest) to 10 (highest) and summed to give a total walkability index score for each CCD. The walkability index scores ranged from 3 to 30 and we used tertile splits (low, medium, high) to categorise CCDs to enable comparison with the Marshall et al. [6] Vancouver study. For more detail on the method of calculation of the walkability index the reader is referred to Mayne et al. [21]. An abridged three-component walkability index was used as data on the fourth factor (retail floor space) [5], was not available for all Sydney CCDs. This abridged three component Sydney walkability index was found to retain 87 % of the variability of a full four factor index [21].

The validity of this walkability index has been previously reported in Mayne et al. [21] using data on “walking entirely to work” reported in the 2006 Australian Census. Mayne et al. [21] found a highly significant exposure-response relationship between walkability and prevalence of walking to work, after adjusting for SES covariates (adjusted odds ratios of: 1.05 (0.96–1.15) for medium walkability; 1.58 (1.45–1.71) high walkability, and 3.02 (2.76–3.30) for very high walkability areas, compared to low walkability areas. This association was similar for the abridged and full walkability index. Furthermore, higher prevalence rates of walking were seen in areas with high walkability, regardless of low or high income grouping; 3.0 % of people walked to work in low income-low walkability areas versus 7.9 % in low-income-high walkability areas; 2.1 % walked to work in high income-low walkability areas versus 11 % in high income-high walkability areas.

We used the 2006 Census data on “walking entirely to work” to determine whether utilitarian walkability behaviour varied according to the potential for TRAP exposure. That is, we determined whether there was a greater proportion of residents walking to work in high walkability areas exposed to high WRD compared to low WRD.

#### Traffic related air pollution

We used a previously developed measure, weighted road density (WRD), as a proxy measure of TRAP [15]. At the time of analysis, there was no Sydney metropolitan wide land use regression model for NO_2_, and the available dispersion model for NO_2_ had a spatial resolution too large (2 x 2 km grid) to reflect the fine spatial differences in this pollutant. We determined the WRD for each CCD (WRD_CCD) (metres per km^2^) using the sum of the weighted road length (metres) divided by the total area enclosed by the CCD boundary (km^2^). The three-tiered weighting system assigned local roads a weight of one, distributor roads a weight of two and motorways, arterial roads and primary roads a weight of three. Digitised road maps (StreetPro Australia Navigation network) [23] were used for road classifications and lengths.

It has been previously shown in Sydney that WRD within a 75 m buffer best predicted measured NO_2_ over alternative buffers ranging from 50 to 400 m [15]. A 75 m buffer is also consistent with evidence of a rapid decline in NO_2_ within the first 75–100 m from a major road [12, 13]. In this study, we averaged WRD across each CCD (WRD_CCD), rather than by a radial 75 m buffer, to match our CCD measure of walkability.

To determine how well the WRD measure correlated with actual NO_2_ measurements, we calculated NO_2_ (annual average and annual average daily maximum concentrations) for 2007 for CCDs: 1) at each of ten regulatory fixed site monitors; 2) within a 200 m radius of each of the ten monitors; 3) within a 2 km radius of each of the ten monitors. It should be noted that the ten monitors were sited in background (non-hotspot) locations in Sydney. We calculated Pearson’s correlation coefficients to determine the correlation between WRD and monitored NO_2_.

### Intersecting walkability and traffic related air pollution measures

We followed the method of Marshall et al. [6] and overlaid tertiles of walkability and WRD to define *“sweet-spots”* (high walkability and low WRD) (desirable), and *“sour- spots*” (low walkability and high WRD) CCDs (undesirable). We also defined CCDs as *“high-spots”* (high walkability and high WRD) and *“low-spots”* (low walkability and low WRD), both health-limiting given their either high WRD or low walkability status. Although the use of tertile combinations is somewhat arbitrary, this method enabled comparison with the Vancouver study. Percentages of neighbourhoods and population sizes within the study area are provided for comparative rather than absolute purposes.

Given that both the walkability and WRD measures use the road structure as inputs for calculation of each measure, we calculated the correlation between walkability and WRD, as well as the correlations between their inputs (residential density, intersection density, land use) respectively. WRD versus walkability was also plotted to visualise the relationship between the two constructs.

### Socio-economic status

For each CCD we used the SEIFA (Socio-economic Indexes for Areas) Index of Relative Socio-economic Disadvantage (IRSD) from the ABS 2006 Census as the measure of socio-economic status (SES) [24]. The IRSD index summarises 17 measures such as income, education and unemployment from data collected in the five-yearly ABS Census. The IRSD scores for the study area were divided into quintiles where quintile 1 represents the 20 % most disadvantaged neighbourhoods (CCDs) (lower SES), and quintile 5 represents the 20 % least disadvantaged neighbourhoods (CCDs) (higher SES).

IRSD quintiles were used to compare the distribution of walkability tertiles and WRD tertiles across quintiles. Prevalence rates (proportions) of the various walkability and WRD categories were calculated by dividing each IRSD quintile proportion by the overall proportion of the walkability-WRD attribute for the whole Sydney metropolitan study area, similar to the method used by Marshall et al. [6]. A ratio of 1 indicates that the relative prevalence of that attribute was the same as the overall prevalence rate, while a score less than 1 indicates a lower proportion compared to the overall rate and vice versa. Bar graphs were also prepared to visualise the relationship between: WRD tertiles and SES; and walkability tertiles and SES.

### Statistical analysis

We used ArcGIS 10.01 Geographical Information System (GIS) software [25] with transformation from Geocentric Datum of Australia 1994 to MapGrid of Australia 1994 Zone 56 for all GIS processing. All statistical analyses and calculations outside the GIS were carried out using SPSS version 21 (Chicago, SPSS, Inc.). Bubble plots were used to display the proportions of CCDs, study population, lowest and highest SES quintiles, and residents walking entirely to work, for each of the walkability-WRD tertiles.

## Results

Walkability scores were calculated for 5858 CCDs (99.5 %) in the study area with 32 CCDs (0.5 %) excluded due to missing data. A WRD score of 0, indicating an absence of road segments, occurred in nine out of 5890 CCDs (0.2 %). Table 1 presents descriptive statistics for both walkability and WRD tertiles.

**Table 1:** Descriptive statistics for walkability and weighted road density for Sydney, 2007

Measure	Statistic	Overall	Tertiles		
			Low	Medium	High
Walkability (range 3–30)	Mean (SD^a^)	16.5 (6.9)	9.4 (2.9)	16.4 (1.7)	24.6 (3.3)
	Median	16	10	16	25
	IQR^b^	11–21	7–12	15–18	21–27
	Min-Max	3–30	3–13	14–19	20–30
	N	5858	2126 (36.3 %)	1834 (31.3 %)	1898 (32.4 %)
Weighted road density (m/km^2^)	Mean (SD)	0.029 (0.012)	0.009 (0.003)	0.016 (0.002)	0.030 (0.015)
	Median	0.016	0.010	0.016	0.027
	IQR	0.012–0.022	0.007–0.012	0.015–0.018	0.022–0.033
	Min-Max	0.000–0.253	0.000–0.013	0.013–0.020	0.020–0.253
	N	5881	1954 (33.2 %)	1964 (33.4 %)	1963 (33.4 %)

^a^SD: Standard deviation

^b^IQR: Inter quartile range

Pearson’s correlation coefficients for the WRD and measured NO_2_ were high and ranged from 0.81 to 0.93 (Table 2). The annual average mean for NO_2_ across the 10 Sydney regulatory monitoring sites was 9.3 ppb (SD: 5.3) and ranged from 5.5 to 13.1 ppb by site. The annual average of the daily NO_2_ maxima across the 10 sites was 19.2 ppb (SD: 8.8) and ranged from 13.1 to 24.6 ppb by site. The scatterplots of WRD versus the three measures of NO_2_ illustrate a tendency for NO_2_ to increase with increasing WRD for both annual average NO_2_ concentration and the annual daily 1 h maximum NO_2_ concentration (Additional file 1: Figure S1).

**Table 2:** Pearson’s correlation coefficients for weighted road density (WRD)^a^ and measured NO_2_ at regulatory monitors

	Corrrelations (significance value)		
Measured monitored site^b^	WRD at Site CCD	WRD 200 m buffer (mean)	WRD 2 km buffer (mean)
Annual average (24 h mean) NO_2_	0.92 (*p* < 0.001)	0.73 (*p* = 0.027)	0.79 (*p* = 0.007)
Annual average 1 h max NO_2_ (mean)	0.86 (*p* < 0.001)	0.79 (*p* = 0.012)	0.83 (*p* = 0.003)

^a^WRD calculated as the mean of WRD of CCDs: 1) at monitored site; 2) within a 200 m radius of each of 10 regulatory monitors; 3) within a 2 km radius of each of 10 regulatory monitors

^b^Measured NO_2_ (2007) at monitored sites: 1) Annual average (24 h); 2) Annual average (1 h maximum) measured at 10 regulatory monitors

Table 3 shows the correlation coefficient for walkability and WRD was 0.52 which was statistically significant. However, walkability was much more highly correlated with its individual components (residential density (*r* = 0.88), intersection density (*r* = 0.80) and land use (*r* = 0.71)), than with WRD. The scatterplot of WRD versus walkability (Additional file 2: Figure S2) demonstrates greater variability in the highest category of WRD (representing major roads and highways), compared to the lowest category (representing relatively quiet back streets).

**Table 3:** Pearson’s correlation coefficients for walkability, weighted road density (WRD), and inputs to the walkability index (residential density, intersection density and land use)

	Pearson’s correlation coefficient				
	Walkability	WRD	Residential density	Intersection density	Land Use
	*N* = 5858				
Walkability	1	0.52^**^	0.88^**^	0.80^**^	0.71^**^
WRD		1	0.46^**^	0.48^**^	0.29^**^
Residential density	-	-	1	0.66^**^	0.45^**^
Intersection	-	-	-	1	0.26^**^
Land use	-	-	-	-	1

**Correlation is significant at the 0.01 level (2-tailed)

**Fig. 1:**
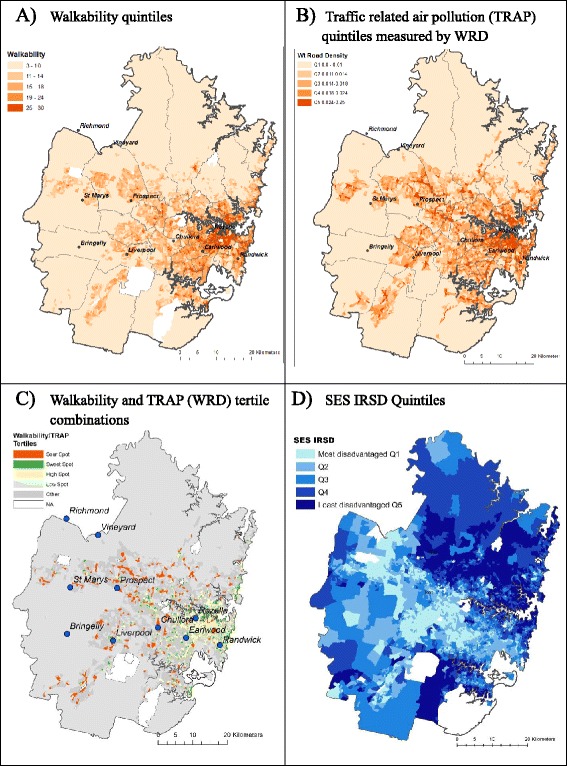
Sydney 2007: (**a**) Walkability quintiles (**b**) Traffic related air pollution (TRAP) quintiles measured by weighted road density (WRD) (**c**) Walkability-TRAP (WRD) tertile combinations – *sweet, sour, high, low-spots* (**d**) SES (Index of Relative Socio-economic Disadvantage (IRSD)) quintiles

Figure 1a and b presents maps of the Sydney metropolitan study area, for walkability and WRD respectively, where darker colour indicates higher values. Walkability demonstrated a clear east-west gradient (Fig. 1a). High walkability was most concentrated in eastern and in western and outer suburbs. High WRD was also concentrated in central Sydney with western Sydney showing more dispersed areas of high WRD corresponding with major suburban centres. These patterns are representative of the Sydney road network which radiates out from the Central Business District (CBD), and associated population densities that are concentrated closer to the CBD and around major suburban centres which are scattered throughout the Sydney metropolitan area.

Nine tertile combinations of walkability and WRD were obtained for the available 5850 CCDs. Four combinations of walkability-WRD are presented in Fig. 1c, representing “*sweet-spots”* (high walkability-low WRD) in green, “*sour-spots”* (low walkability- high WRD) in orange, “*high-spots”* (high walkability-high WRD) in cream, and “*low-spots”* (low walkability-low WRD) in light grey. The remaining five tertile combinations are presented as one category “*Other*” (in dark grey).

**Fig. 2:**
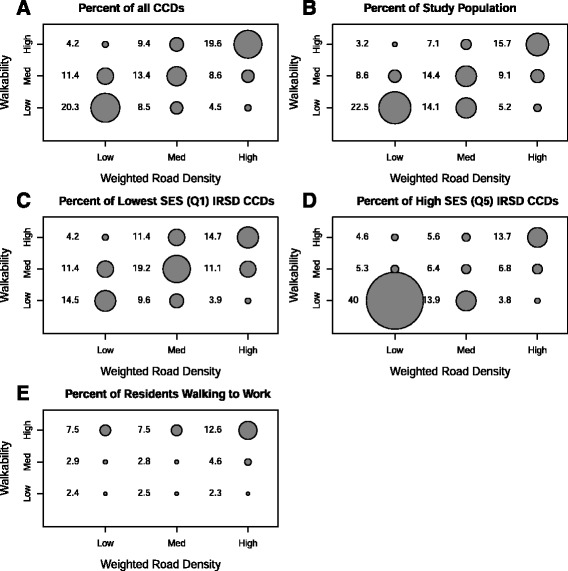
Characteristics in each Walkability-WRD tertile (%): (**a**) Percent of CCDs in overall study area; (**b**) Percent of study population; (**c**) Percent lowest SES (IRSD) quintile (most disadvantaged; stratified analysis); (**d**) Percent highest SES (IRSD) quintile (least disadvantaged; stratified analysis); (**e**) Percent of residents walking to work (this plot represents the sub-group of residents living in the study area who “walked entirely to work”, as reported in the 2006 Census, Australian Bureau of Statistics). TRAP measured by weighted road density (WRD)

Figure 2, Panel a shows the percentage of CCDs in each of the nine walkability-WRD tertiles (if walkability and TRAP were not correlated, each tertile combination would be 11 %). A minority of CCDs were either “*sweet-spots”* or “*sour-spots”*. A total of 245 (4.2 %) CCDs were “s*weet-spots”* with high walkability score (mean = 23.3, SD = 2.8, range 20 to 30) and low WRD (mean = 0.010, SD = 0.003, range 0.001 to 0.013). A similar number of CCDs (*n* = 265, 4.5 %) were classified as “*sour-spots”* with a low walkability score (mean = 11.0, SD = 1.8, range 4 to 13) and high WRD (mean = 0.025, SD = 0.006, range 0.019 to 0.05). This corresponded to 3.2 % (*n* = 115,069 persons) of the study population living in “*sweet-spots”* (summed over “*sweet-spot*” CCDs), and 5.2 % (*n* = 188,916 persons) living in “*sour-spots”* (summed over “*sour-spot*” CCDs) (Fig. 2, Panel b). Table 4 indicates that the relative population prevalence is lower than expected for “*sweet-spots”* and higher than expected for “*sour-spots”* based on the proportions of CCDs compared with all CCDs overall.

**Table 4:** WRD and walkability tertiles: relative prevalence^a^ for population and by IRSD quintiles^b^, 2007

Measure		All CCDs ^c^ (100 %)	Low WRD	Low Walk	Mid WRD	Mid Walk	High WRD	High Walk	Sweet -Low WRD -High Walk	Sour -High WRD -Low Walk	High -High WRD -High Walk	Low -Low WRD -Low Walk
Proportion of CCD’s (%)		100	33.0	36.3	33.5	31.4	33.5	32.3	4.2	4.5	19.6	20.3
Population (Relative prevalence)		1.00	1.04	1.15	1.07	1.03	0.90	0.80	0.76	1.15	0.80	1.11
IRSD Category	1 low SES	1.00	0.92	0.76	1.18	1.32	0.90	0.96	1.06	0.85	0.77	0.72
	2	1.00	0.82	0.79	1.08	1.24	1.09	1.00	0.85	1.16	1.04	0.63
	3	1.00	0.89	0.86	1.01	1.10	1.10	1.07	0.81	0.98	1.15	0.82
	4	1.00	0.86	1.00	0.96	0.76	1.18	1.23	1.18	1.16	1.34	0.85
	5 high SES	1.00	1.51	1.59	0.77	0.59	0.72	0.74	1.10	0.85	0.70	1.97

^a^Values in each column represent the relative prevalence of IRSD within each category normalised to 1.0 (being equal to the overall prevalence of IRSD category across the Sydney metropolitan area). That is, a value of 1.51 for low WRD in the highest (5) SES category represents a 51 % higher than expected prevalence of low WRD CCDs compared with low WRD across all SES categories/all CCDs. A value of 1.18 for *sweet-spot* CCDs in the second-highest (4) SES category represents a 18 % higher than expected prevalence of *sweet-spot* CCDs compared with *sweet-spots* across all SES categories/all CCDs. A value of 0.76 for low walkability in the lowest (1) SES category represents a 24 % lower than expected prevalence of low walkability CCDs compared with low walkability across all SES categories/all CCDs

^b^IRSD-Index of Relative Socio-economic Disadvantage sourced from the Australian Bureau of Statistics 2006 Census. IRSD is used as the measure of area-based SES in this analysis

^c^CCD-Census Collector District-smallest geographical unit for which walkability and weighted road density (WRD) were calculated

Compared to “*sweet-spots*” and “*sour-spots*”, substantially more CCDs were either *“high-spots”* ((high walkability-high WRD) (*n* = 1147 (19.6 %), walkability score: mean = 25.4, SD = 3.3, range 20 to30; WRD score: mean = 0.033, SD = 0.017, range 0.020–0.252), or “*low- spots”* (low walkability-low WRD) (*n* = 1188 (20.3 %); walkability score: mean = 8.4, SD = 3.0, range 3–13; WRD score: mean = 0.008, SD = 0.003, range 0.080–0.133) (Fig. 2, Panel a).

High walkability CCDs had the greatest proportions of people walking to work, with almost double the proportion (12.6 %) walking to work in high WRD CCDs than in low or medium WRD CCDs (7.5 % each) (Fig. 2, Panel e). These proportions were double those in medium or low walkability CCDs.

**Fig. 3:**
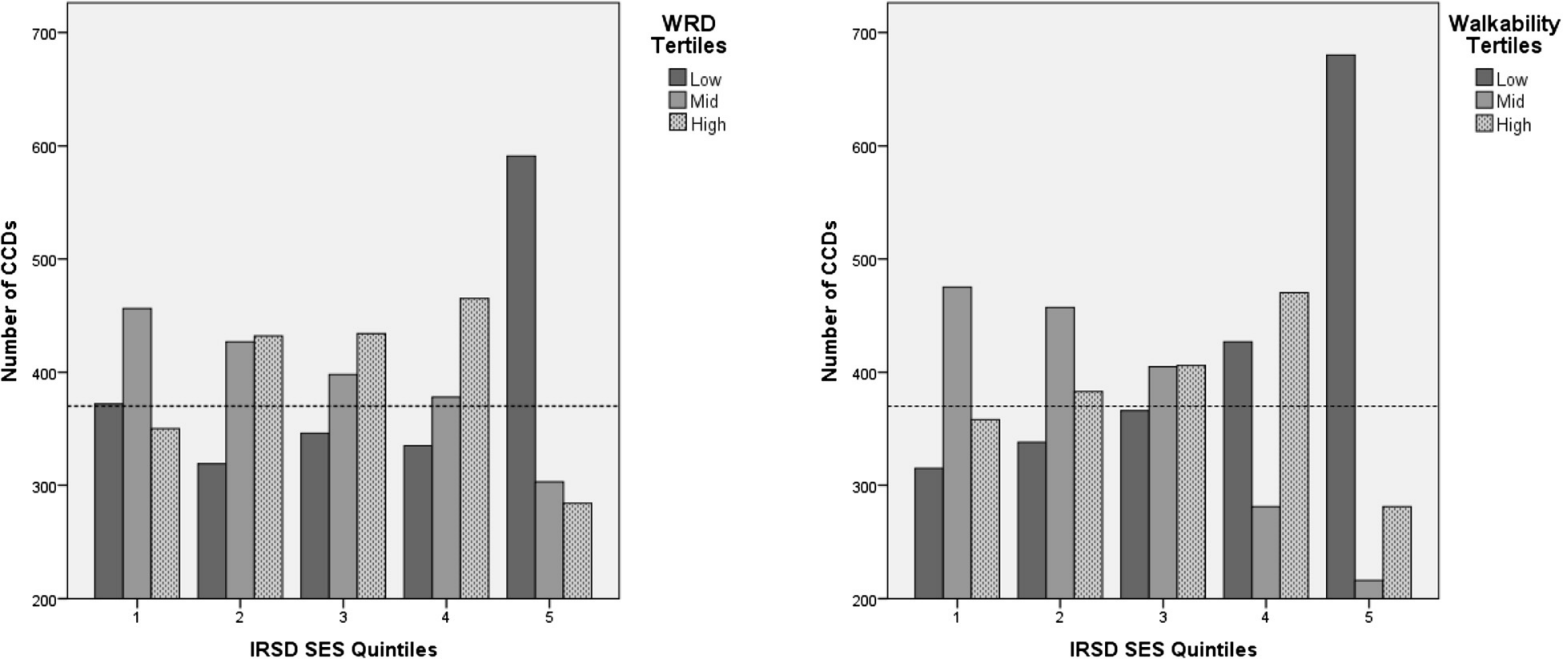
CCDs by IRSD (SES) quintiles for WRD tertiles and for walkability tertiles. The horizontal line represents the expected number of CCDs if there was no association between SES (IRSD) quintiles and levels of WRD and walkability

The relationships between: WRD tertiles and SES; and walkability tertiles and SES are shown in separate bar graphs in Fig. 3. The horizontal line represents the expected number of CCDs if there was no association between SES quintiles and levels of WRD or walkability. The charts show no clear relationship between WRD and SES or between walkability and SES. However, the number of CCDs within the high WRD tertile tended to increase with increasing SES quintiles, until the highest quintile where there was a marked decrease in the number of CCDs with high WRD. The mid-WRD tertile had a tendency to decrease with increasing SES quintiles ie decreased with lower disadvantage. The bar chart also illustrates the much higher proportion of CCDs with low WRD in the highest SES quintile, contributing to the much higher than expected prevalence of *“low-spot”* CCDs in the highest SES quintile (see below). There was no clear relationship between walkability and SES, except that the number of CCDs with low walkability increased with increasing SES. High walkability also increased with increasing SES, except for the highest SES quintile, where the number of high walkability CCDs was substantially lower than for all other SES quintiles.

Figure 2, Panels c and d show the proportions of the walkability-WRD tertile combinations for the lowest (1^st^, most disadvantaged) and highest (5^th^, least disadvantaged) SES quintiles respectively. Table 4 summarises the relative prevalence of *sweet-, sour-, high-* and *low*- spot CCDs by SES quintiles. The proportion of *sweet-* and *sour-spot* CCDs in both the lowest and highest SES quintiles were similar at around 4 % (Fig. 2, Panels c and d). There was a higher prevalence of “*sweet-spots”* in the two highest SES quintiles (least disadvantaged), with a non-linear relation across the five SES quintiles (Table 4). There were no clear trends evident for *“sour-spots”* or *“high-spots”* across SES quintiles, although both the highest and lowest SES quintiles had a lower relative prevalence of *“sour-spots”* (0.85, 0.85 respectively) and *“high-spots”* (0.70 and 0.77 respectively) and similar percentages of *“high-spot”* CCDs (14.7 % in the lowest and 13.7 % in the highest SES quintiles) (see Fig. 2, Panels c and d).

The middle SES quintiles had a higher relative prevalence of high walkability-high WRD. The proportion of *“low-spot”* CCDs (low walkability-low WRD) in the highest SES quintile (40.0 %) was more than double that in the lowest SES quintile (14.5 %) corresponding to a much higher prevalence of *“low-spot”* CCDs in the highest SES quintile (1.97), with lower than expected prevalence for the four other SES quintiles (Table 4).

## Discussion

Our study explored spatial patterns of, and association between, walkability (for utilitarian purposes) and TRAP (using WRD as a proxy) in the Sydney Metropolitan area.

Our maps of walkability and WRD identified “*sweet-spot*” neighbourhoods where the built environment (specifically land use and street networks) is conducive to good population health, and “*sour-spot*” neighbourhoods where the built environment is detrimental to population health, as well as identifying *“high-spots”* (high walkability-high WRD) and *“low-spots”* (low walkability-low WRD). These maps and associated analyses by SES can help identify areas that are subject to environmental inequalities with respect to representing health-promoting or health-limiting environments which could benefit from targeted urban and transport planning and, public health interventions. In further work, we will extend our analyses to include investigations of the association between walkability, weighted road density and health outcomes.

The maps can also help identify neighbourhoods that could be potentially transformed into *“sweet spots*” through redevelopment efforts, such as infill development and traffic calming. Overall, this methodology for mapping and describing spatial interactions of walkability and WRD represents a useful tool for informing urban planning and transport policy initiatives to improve neighbourhood walkability while reducing, or at the very least, not worsening TRAP.

Overall our findings are similar to the Vancouver results by Marshall et al. [6] for patterns of walkability and TRAP. We found a very small proportion of Sydney neighbourhoods (4.2 %) classified as *“sweet-spots”*, remarkably similar to the Vancouver study (3.6 % of postcodes) [6]. Sydney’s 4.2 % “*sweet-spot”* neighbourhoods represent 3.2 % of the actual population, and while this difference of -1 % is small, multiplied across the Sydney population, it corresponds to 37,000 people who do not benefit from walkability-TRAP environments that are conducive to good health.

“*Sweet-spots”* and “*sour-spots”* occurred throughout the Sydney metropolitan region. As with Vancouver, we found *“sweet-spots”* to be located near, but not in the city centre CBD, and were more prevalent in the highest SES quintiles. Not surprisingly, *“sweet-spot”* neighbourhoods were mostly found along the harbour foreshore and coastal strip, higher population density areas, which are also highly desirable residential areas in Sydney. The 4.5 % of *“sour-spot”* neighbourhoods (5.2 %; 187,200 of the population) in our study, similar to Vancouver (6.8 %), were scattered widely, at distance from the Sydney CBD, more distant from the harbour or coastal areas, and more prevalent in the middle SES quintiles. “*High-spots”* tended to be aggregated closer to the Sydney CBD. “*Low-spots”* were primarily located in lower density residential regions around the outer perimeter of the Sydney metropolitan area.

We found no clear trends in the distribution of walkability or TRAP by neighbourhood-level SES. Low WRD was mainly observed in the highest SES neighbourhoods in Sydney (1.51). High WRD occurred mainly in the middle SES neighbourhoods, with the highest SES neighbourhoods having the lowest prevalence (0.72). Although there were no linear relationships between WRD and SES in Sydney, the findings that low WRD was substantially more prevalent, and high WRD had the lowest prevalence, in the highest SES neighbourhoods, suggests some environmental inequality in the distribution of TRAP. However, low walkability was also more prevalent in the highest SES neighbourhoods in Sydney, suggesting that opportunities to improve walkability also exist for high SES neighbourhoods. While there was a non-linear relationship between high walkability and SES, the second highest SES category had the highest relative prevalence of walkability (1.23).

The Sydney findings vary from the Vancouver study which reported a linear association between low NO pollution and SES category in Vancouver (0.55 to 1.42) and an inverse linear association between high NO pollution and SES (1.55 to 0.69) [6]. A recent Australian study investigating environmental inequality of NO_2_ in urban areas, reported that NO_2_ concentrations decreased in areas with less disadvantage, however, the actual differences in concentrations was very small at 0.8 ppb [26]. As indicated in overseas studies, the relation between air pollution and SES can be complex and not always in the expected direction [16, 19]. Our findings indicate that there are opportunities for improved walkability across all SES settings.

We found similarly large proportions of *“high-spots”* (high walkability/high WRD) and *“low-spots*” (low walkability/low WRD) in around 20 % of Sydney CCDs. If walkability and TRAP were independent of each other, we would expect 11 % each of CCDs in *“high-”* and *“low-spots*”, which is half of the observed percentages. *“High-spots”* tended to be located in population dense areas closer to the Sydney CBD while *“low-spots”* tended to be located towards the perimeter of the Sydney metropolitan region bordering national parks, recreational reserves, government and farming land. This pattern is indicative of the Sydney road network which radiates out from the more densely populated central and eastern suburbs around the harbour and coastal fringe. This pattern of land use may be particularly susceptible to health trade-offs between walkability and exposure to TRAP [27].

We know from previous work, that the Sydney Walkability Index used for this study correlates well with measures of walking in Sydney [21]. Our analyses reported a two to three -fold increase in the proportion of people walking entirely to work in high walkability CCDs compared to medium or low walkability CCDs. Perhaps most importantly, our study also found that the proportion of residents walking entirely to work in high walkability CCDs located in high WRD areas was almost double than for high walkability CCDs located in low WRD areas. This finding suggests that people do not currently modify their walking patterns based on knowledge or assumptions of neighbourhood air quality, and thus opportunities exist to minimise air pollution exposure while not discouraging walking.

Our study has several important policy implications. First, it identifies geographical areas in Sydney that are exposed to higher environmental hazards in terms of low walkability and high TRAP. Populations living in *“sour spots”* are likely to suffer from added disease risks from physical inactivity and air pollution [27]. This likely reflects an environmental injustice that requires policy actions in terms of targeted programs and distribution of resources to reduce health inequality. We found that the annual mean NO_2_ concentrations (and annual average daily 1 h maximum NO_2_ concentrations) were 9.3 ppb (19.4 ppb) across the ten monitored sites in Sydney, with a range of 5.5–13.1 (13.1–24.6) ppb, depending on site location. This represents an almost two-fold variation in pollutant concentrations. The NSW regulatory standard for 1 h maximum NO_2_ is 120 ppb, and while the highest average daily 1 h maximum concentration was 55 ppb, it should be noted that all of the monitors were sited in background rather than hot-spot locations. Thus it is likely that much higher readings would occur in heavily trafficked locations, demonstrating the opportunities for exposure minimisation.

Second, this study revealed that walkable areas where people are more likely to participate in active transport, such as walking and cycling, tend to have higher TRAP. This finding is potentially important in guiding planning initiatives regarding the locations of pedestrian and cycling infrastructure. Previous studies have shown that concentrations of air pollutants can vary depending on the route chosen, with quieter or dedicated cycling/walking routes associated with lower TRAP exposures for runners [28] and cyclists [29–31]. TRAP exposures also vary over even smaller spatial scales, with studies reporting lower pollutant exposures (measured/modelled) for: pedestrians compared to in-vehicle exposures [32]; pedestrians walking closer to building envelopes than the road kerbside [33]; and a pedestrian boardwalks separated from the roadside [34]. Clearly, improving neighbourhood walkability without a detrimental increase in TRAP exposure will require re-examination of where pedestrian footpaths and bike lanes are placed, with several studies calling for greater separation between vehicles and pedestrians/cyclists [28, 35, 36]. Planning and building active transport infrastructure with these points in mind could maximise health gains from increasing/improving walkable/cyclable neighbourhoods without compromising exposure to air pollution.

The third and most upstream policy implication of our study, and one relevant to many international cities, is providing insight into future transport planning and development initiatives to design or transform neighbourhoods to be walkable while ensuring low exposure to TRAP. In 2013, the NSW state government released a strategy for improving walkability, with particular attention placed on Sydney. While the strategy highlights many examples, it focuses on increasing the number of walking trips per person within 2 km of a destination or urban centre [37]. At the same time there is debate over major urban transport projects and integrated land use [38]. For example, the proposed Sydney WestConnex scheme is a 33 km road infrastructure project linking sections of Sydney through a series of road tunnels and includes urban renewal of a currently heavily trafficked surface road west of the city centre, primarily a high walkability/high TRAP location. This scheme could provide a major opportunity to incorporate planning measures which improve walkability, urban connectedness and reduce TRAP, if coupled with a reduction in vehicular use [39], and increased active or public transport infrastructure, and improved land use mix. A North American study showed that walkability, mixed land use, better street connectivity and higher population densities are associated with smaller but significantly lower estimated NOx concentrations [40]. This demonstrates that reducing localised TRAP requires a critical rethink of how we plan urban re/development and transportation systems.

Frank and Engelke (2005) have also highlighted the need for other multi-component strategies to achieve better walkability while maintaining or reducing TRAP emissions, including altering utility across different travel modes within neighbourhoods so that motorised modes are made less attractive and active travel options become a safer and more attractive experience, focussing on green technologies for motor vehicles or using economic disincentives such as parking fees or zones to discourage motor vehicular use [41].

A strength of our study is the use of previous methodology, and although the use of tertiles for defining walkability-WRD categories is arbitrary, it enabled comparisons to be made between two very different settings-Sydney and Vancouver [6]. While the use of quartiles or quintiles would have resulted in different proportions of *sweet-* and *sour-spots*, the resultant increased number of categories could have made comparisons unwieldy.

We used a previously validated measure of walkability that was associated with walking to work in Sydney [21]. Our estimate of TRAP applied a WRD measure that was validated for an area within Sydney where it explained 59 % of the variability in roadside NO_2_, a commonly used marker of TRAP [15]. We also found high correlations between WRD for the CCD at the air quality monitoring site (and for CCDs within 200 m and 2 km of the monitors) and annual averaged NO_2_ measurements from those monitors (spread across the Sydney metropolitan area), suggesting that WRD, our proxy for TRAP, is a valid measure for this analysis. Improvements in our methodology might include the use of land use regression or dispersion modelled NO_2_ estimates as they become available for the study area [42].

The underlying input of the road network to the calculations of both walkability and WRD is another potential limitation and might partly explain the large percentage of low-low and high-high walkability-WRD observations. However, walkability was less strongly correlated to WRD than it was to its input components of residential density, land use and intersection density, suggesting that other features such as residential density and land use are important in determining walkability opportunities in urban settings.

A limitation of our study was the use of a variable sized spatial unit–the CCD. Australia introduced a new standard geography for census data reporting in 2011 including substantially smaller spatial units (Mesh Blocks) than CCDs and future work could assess the sensitivity of our walkability-WRD distributions to this smaller spatial unit [43]. Despite the variability in the size of our spatial unit compared to the average size of Vancouver postcodes of 0.05 km^2^, the results for overall population and neighbourhoods deemed *“sweet-spots”* and *“sour-spots”* were highly consistent.

## Conclusions

This study found that few neighbourhoods in Sydney have health promoting attributes of both high walkability and low TRAP, while much larger proportions of neighbourhoods have health limiting attributes of high TRAP exposures or low walkability. Of concern, over five percent of the Sydney population lives in neighbourhoods which have both low walkability and high TRAP, thus subject to a double burden of environmental attributes conducive to poor health outcomes.

To remedy this situation, state and local governments should seek out opportunities for increased neighbourhood walkability through improved urban development and transport planning, taking care that new infrastructure projects, in-fill and redevelopments do not result in a concomitant increase in TRAP exposure, especially amongst highly exposed groups in the population like pedestrians in high traffic areas and within lower SES neighbourhoods.

## Additional files

Additional file 1: Figure S1. Scatter plots of a) NO_2_ annual daily average and b) NO_2_ Annual average daily 1 h maximum, with WRD at monitored sites, within a 200 m radius of monitored sites and within a 2 km radius of monitored sites. (PDF 175 kb) Additional file 2: Figure S2. Scatterplot of walkability versus weighted road density (WRD). (PDF 166 kb)

## Abbreviations

ABS: Australian Bureau of Statistics
CBD: Central Business District
CCD: census collector districts
GIS: geographic information system
IRSD: Index of Relative Socio-economic Disadvantage
NO: oxides of nitrogen
NO_2_: nitrogen dioxide
NSW: New South Wales
ppb: parts per billion
SD: standard deviation
SES: socio-economic status
TRAP: traffic related air pollution
WRD: weighted road density
