# Supplementary figures and images for: Neighbourhood walkability, road density and socio-economic status in Sydney, Australia
Source: Environ Health. 2016 Apr 27;15:58. doi: 10.1186/s12940-016-0135-y (PMC4847364; doi:10.1186/s12940-016-0135-y)

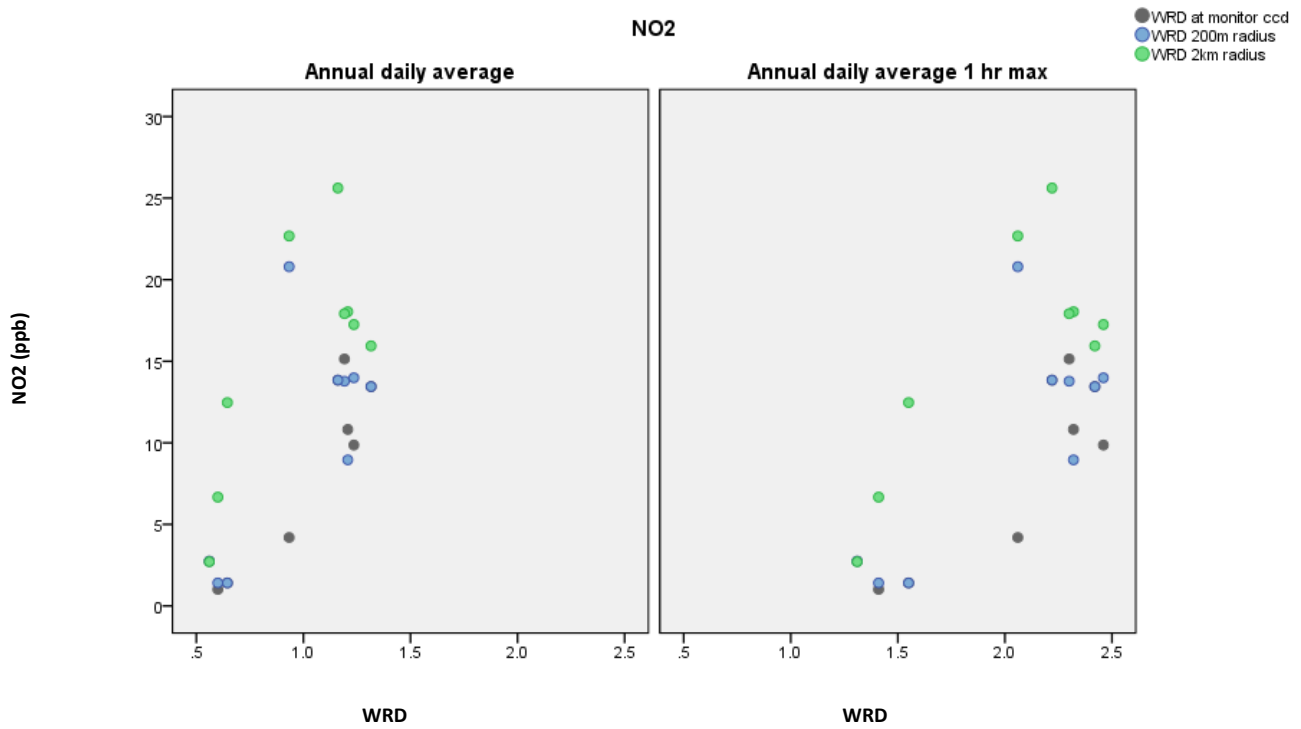

Supplement: Additional file 1: Figure S1. — Scatter plots of a) NO2 annual daily average and b) NO2 Annual average daily 1 h maximum, with WRD at monitored sites, within a 200 m radius of monitored sites and within a 2 km radius of monitored sites. (PDF 175 kb) [file 12940_2016_135_MOESM1_ESM.pdf]

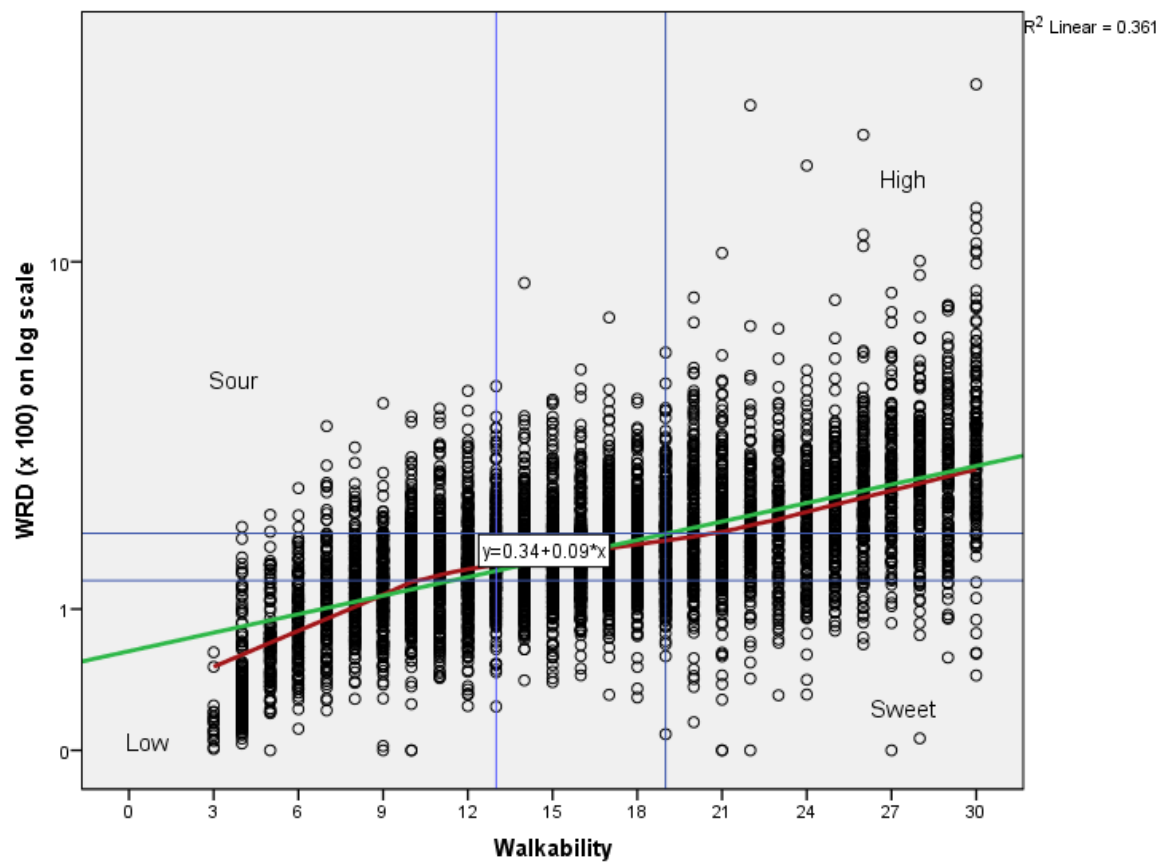

Supplement: Additional file 2: Figure S2. — Scatterplot of walkability versus weighted road density (WRD). (PDF 166 kb) [file 12940_2016_135_MOESM2_ESM.pdf]
